# Australasian Society of Clinical Immunology and Allergy consensus statement on IEI molecular diagnosis

**DOI:** 10.70962/jhi.20250250

**Published:** 2026-06-16

**Authors:** Tatiane Yanes, Theresa Cole, Alisa Kane, Jovanka King, Alberto Pinzon-Charry, Kuang-Chih Hsiao, Peter McNaughton

**Affiliations:** 1 Frazer Institute, The University of Queensland, Brisbane, Australia; 2 Queensland Paediatric Immunology & Allergy Service, Queensland Children’s Hospital, Brisbane, Australia; 3 Infection and Immunity, Murdoch Children’s Research Institute, Melbourne, Australia; 4 Department of Pediatrics, University of Melbourne, Melbourne, Australia; 5 Department of Clinical Immunology and HIV, St Vincent’s Hospital Sydney, Darlinghurst, Australia; 6 Department of Clinical Immunology and HIV, Liverpool Hospital, Liverpool, Australia; 7 St Vincent’s Health Care Campus, School of Clinical Medicine, Faculty of Medicine and Health, UNSW Sydney, Sydney, Australia; 8 Women’s & Children’s Health Network, SA Pathology, Adelaide University, Adelaide, Australia; 9 Griffith University, Brisbane, Australia; 10 Department of Paediatrics Child and Youth Health, University of Auckland, Auckland, New Zealand; 11 Department of Immunology, Starship Children’s Hospital, Te Whatu Ora, Auckland, New Zealand; 12 Department of Paediatrics and Child Health, The University of Queensland, Brisbane, Australia

## Abstract

Genomic testing has transformed the diagnosis and management of inborn errors of immunity (IEIs). Despite its rapid uptake, there remains limited guidance for clinicians on which patients should undergo testing. The Australasian Society of Clinical Immunology and Allergy (ASCIA) has developed evidence-based guidelines to support clinicians in identifying individuals who may benefit from genomic testing for suspected IEI. This guideline paper reviews current literature and reports expert consensus to provide practical recommendations on patient selection, testing modalities, and interpretation of results. It outlines clinical scenarios where genomic testing is most likely to yield actionable insights, including early-onset, severe, or atypical immune presentations and familial patterns suggestive of heritable immune dysfunction. The ASCIA guidelines aim to support genomic testing decision-making and ultimately improve diagnostic accuracy, access to timely interventions, and outcomes for individuals with IEI across Australasia and internationally.

## Teaser

Yanes et al. presents evidence-based guidelines from the Australasian Society of Clinical Immunology and Allergy to support clinicians in selecting patients with suspected inborn errors of immunity for genomic testing. The work provides practical recommendations to improve clinical decision-making, delivery of diagnostic genomic testing, and patient outcomes.

## Introduction

Over the last 30 years the rapid increase in the understanding of the genetic basis of human inborn errors of immunity (IEIs) has led to significant improvements in diagnosis, treatment, and quality of life of patients with IEI, including primary immunodeficiencies (PID) (1, 2). These recognized benefits, along with increased access to and decreased cost of genomic testing, have led to a rapid increase in utilization of these tests. Genomic testing has also become important earlier in the diagnostic pathway and is increasingly requested by nongeneticists in mainstream care (3). The potential benefits of genomic testing for this patient group are well recognized and includes clinical, economic, and patient reported outcomes (4, 5). The use of genomic testing by clinical immunologists is supported by international peak bodies (2).

Despite the rapid increase in genomic testing use for IEI, there are limited guidelines to inform which patients should be offered testing (6), with available guidelines limited to specific IEI such as monogenic forms of inflammatory bowel disease (IBD) (7, 8). Use of genomic testing has significant implications for patients, families, and health budgets and requires careful consideration of the risks and benefits for each patient (9). This document was developed by the Australasian Society of Clinical Immunology and Allergy (ASCIA) to help guide clinicians in making decisions about appropriate genomic testing for patients with an IEI. To ensure the recommendations were evidence-based and clinically relevant, ASCIA convened an expert advisory, which conducted a literature review and applied expert consensus to formulate practical guidance on patient selection and testing approaches.

## General principles

### Choosing testing modality

There are several genetic testing methodologies, and choice of test modality depends on factors including clinical presentation, the suspected genomic mechanism (e.g., single nucleotide variant, structural rearrangements, copy number variant, or germline or somatic variant), funding availability, access to genomic technology, and clinical and laboratory expertise locally (2, 10, 11). Currently there is no federal-level Medicare funding for IEI genomic testing in Australia. Costs are covered privately by the patient or the state-level Hospital and Health Services.

Sequencing technology vary in their approach and capabilities. Most platforms currently use next-generation sequencing (NGS), which all but replaced Sanger sequencing due to its higher throughput, faster turnaround time, and cost effectiveness (10, 11). Common types of NGS-based testing include targeted single gene or gene panel, whole-exome sequencing, and whole-genome sequencing. Other genomic technologies include microarray-based platforms (e.g., CGH arrays) (11), long-read sequencing (10, 12), and methods for interrogating noncoding regions (e.g., RNA sequencing) (13), with the latter two typically limited to research in Australia. Identification of somatic mutations remains technically challenging, with standard NGS approaches lacking sufficient sequencing depth to reliably detect low-frequency mosaic variants (14). In such cases, targeted deep sequencing may improve detection of somatic variants. Selection of the appropriate sample type is also critical to ensure that the tested tissue contains the somatic variant.

**Recommendation:** NGS is recommended as the initial genetic test for most patients with suspected IEI. Single gene testing should only be considered in patients with clinical phenotypes and functional testing highly specific for a single gene.

Targeted panel testing, including virtual panels derived from WES, is a commonly approach for diagnosing IEI (11). Panels used by different genomic testing laboratories do not all contain the same gene lists. Some panels may not contain all genes known to be associated with a phenotype or may contain genes that have limited evidence to support an association with a phenotype.

There are curated gene panels for phenotypes associated with IEI, such as PanelApp Australia (https://panelapp-aus.org/), which aim to establish an up to date, robust evidence base for gene-disease relationships (15). Some recently reported genes may reach a threshold for testing in research setting but may not yet reach the threshold of evidence for a clinical genetic report or to guide treatment.

**Recommendation:** Clinicians ordering genomic testing should be aware of the genes included in the panel they are requesting, including that the genes associated with the phenotype under investigation have been included and/or whether genes not associated with presentation have been tested.

### Result interpretation

A genetic diagnosis should not be a prerequisite confirmatory test to initiate supportive therapy, and therapy should not be withheld while genomic results are pending (2).

Many genes are associated with highly variable phenotypes, and in most cases, treatment decisions should be based on phenotype and not rely solely on the genetic diagnosis. The decision to offer genomic testing for patients with suspected IEIs should be done in consultation with an immunologist with experience in managing these disorders and interpreting genomic test results.

A genomic test that does not reveal a genetic cause for a patient’s presentation cannot be used to exclude an IEI or a genetic diagnosis. A genetic cause beyond the limits of testing technology or current knowledge remains possible, with a near exponential increase in gene discovery (1). Reanalysis of genomic testing has been shown to increase diagnostic yield (16, 17).

All people have benign variants in their genetic code, including in IEI-associated genes. There are international guidelines, including by the American College of Medical Genetics, that inform laboratory variant classification (18). While some variants are known to be pathogenic or benign, there are many variants with an uncertain contribution to disease, known as variants of uncertain significance (VUS). The chance of discovering a VUS in a patient is increased proportionally to the number of genes tested and is also more likely in patients of non-white European ancestry (19).

VUS should not be used in isolation to make treatment decisions for a patient, as this can lead to inappropriate and harmful treatment (20, 21, 22). Where possible, genomic testing should be targeted to genes known to be associated with the presenting phenotype. When the presenting phenotype is associated with a large number of genes, tiered testing targeting the most likely diagnoses first, before expanding to broader panels, should be considered. With the increasing number of known disease-associated genes and evolving variant interpretation, there is a responsibility to periodically review genomic testing results over time. In some cases, repeated genomic testing or reanalysis may be indicated (23, 24).

### Genetic counselling

Genetic counselling should be provided before and after genomic testing, ideally with both an immunologist and a genetic counsellor with expertise in IEI (9). Standard consent forms and information sheets are now available via Australian Genomics to support pretest discussion with patients (25, 26). Post-results, clinical immunologists who order genomic testing for patients should be prepared to discuss (or refer the patient to a genetic counsellor for discussion of) (1) the nature of test results, (2) the consequences and implications of the condition, (3) the probability of developing additional symptoms, and (4) family planning options (2, 9). There are also several important familial considerations, including identifying at-risk relatives, informing reproductive decision-making, and cascade testing. Despite the potential benefits, cascade testing remains underutilized, even for conditions with strong evidence-based recommendations (27), leading to missed opportunities for early diagnosis, prevention, and reproductive-decision making. This gap highlights the importance of the clinician’s duty of care, not only to the proband, but also to their family, by ensuring that appropriate follow-up, communication, and access to testing are facilitated (Table 1) (28).

**Table 1.** Summary of recommendations

Condition	Genomic testing recommended
SCID	Rapid genomic testing is recommended for all patients with SCID
CID	Genomic testing should be considered in all patients with CID
Phagocytic defects	Genomic testing is recommended for all patients with suspected monogenic phagocytic defects, including CGD and LAD
Susceptibility to specific infections	Genomic testing is recommended for patients with susceptibility to specific organisms suggestive of a monogenic IEI
Evans syndrome	Genomic testing is recommended for all pediatric patients presenting with Evans syndrome
Autoimmune lymphoproliferative syndromes	Genomic testing is recommended in all pediatric patients with autoimmune lymphoproliferative syndromes and should be considered in patients with unexplained chronic or recurrent lymphadenopathy, splenomegaly, or organ infiltration by abnormal lymphoid cells. In the case of a negative result, discussion with a geneticist in regard to somatic testing is recommended
**Condition**	**Genomic testing recommended for some patients**
Immune dysregulation	Genomic testing should be considered in patients with immune dysregulation based on clinical features, including early age of onset, severity of disease, treatment resistance, and family history
HLH	Rapid genomic testing is recommended for all pediatric patients with suspected familial or primary HLH due to treatment implications. The likelihood of a genetic diagnosis in adults with HLH is lower and decisions to proceed with genomic testing should be made in consultation with an adult immunologist or hematologist with expertise in HLH
Very early onset IBD (VEO-IBD)	Genomic testing should be considered for all children presenting with IBD <2yo and for patients >2yo with atypical features, including recurrent infections, complex autoimmunity, severe perianal disease, treatment resistance, and family history
Autoinflammation/periodic fever	Genomic testing should be considered in selected patients who present with a classical phenotype suggestive of a monogenic disorder or based on atypical features, severity of disease, treatment resistance, and family history to guide selection of therapy, especially if associated with early age of onset
Predominantly antibody deficiencies	Genomic testing should be considered in patients with a high suspicion of a monogenic disorder, based on clinical features, including early age of onset, severe disease, and family history

## Summary of recommendations

### Severe combined immunodeficiency

Severe combined immunodeficiency (SCID) is a heterogeneous group of disorders characterized by impaired T cell (T lymphocyte) development (29). Newborn screening for SCID is standard of care throughout Australia and New Zealand. Early definitive treatment is lifesaving for these patients (29).

Approximately 90% of patients with SCID have a monogenic IEI (30). Defining the molecular cause of SCID has important treatment implications with genetic diagnoses that are not correctable with hematopoietic stem cell transplantation (HSCT), such as thymic disorders. Some of these genetic diagnoses require alternative conditioning regimens (e.g., DNA ligase 4) and availability of enzyme or gene therapy for some patients (e.g., ADA deficiency).

**Recommendation:** Rapid genomic testing is recommended for all patients with SCID.

### Combined immunodeficiency

Combined immunodeficiencies (CID) comprise a heterogeneous group of disorders with impaired development, function, or both of T lymphocytes associated with a defective antibody response (31).

These conditions are generally less profound than SCID and can present throughout childhood and adolescence and rarely in adulthood. Some CID present with syndromic features or recognized patterns of associated features. Defining genetic diagnoses for these patients can have important prognosis and treatment implications, including HSCT and specific targeted therapies available for some conditions.

In patients with specific phenotypes or syndromic features, consider targeted testing. In some cases, broad testing of all PID associated genes may be appropriate.

**Recommendation:** Genomic testing should be considered in all patients with CID.

### Predominantly antibody deficiencies

Antibody deficiencies, leading to increased infection susceptibility, are the most common form of PID (31). This heterogenous group of conditions can present at any time during the lifespan and can range in severity from complete absence of immunoglobulin (e.g., X-linked agammaglobulinemia) to less severe phenotypes. There are a number of identified monogenic causes of predominantly antibody deficiencies with important prognostic, surveillance, treatment, and family planning implications; however, many antibody deficiencies remain genetically undefined (32).

The lack of molecular diagnosis makes selection of patients for genomic testing difficult, as there is insufficient evidence to support the routine use of genomic testing in all patients, particularly with late-onset, less severe manifestations. One study demonstrated a diagnostic yield of 27% in patients who met the formal European Society for Immunodeficiencies ePan-American Group for Immunodeficiency diagnostic criteria for common variable immunodeficiency and had at least one of the following additional criteria: disease onset at age <18 years, autoimmunity, low memory B lymphocytes, family history, and/or history of lymphoproliferation (33).

**Recommendation:** Genomic testing should be considered in patients with a high suspicion of a monogenic disorder, based on clinical features, including early age of onset, severe disease, and family history.

### Phagocytic defects

Inherited functional neutrophil disorders can be caused by defects in neutrophil respiratory burst, leukocyte adhesion, and phagocytosis. Some PID can also be associated with neutropenia. These conditions can often be clinically diagnosed using nongenetic functional testing. Characterization of the genetic defect and inheritance pattern can have important implications for testing of family members, family planning, and selection of stem cell donors.

There are several identified genetic causes of isolated congenital neutropenia; however, investigation of these conditions is outside the scope of this guideline.

**Recommendation:** Genomic testing is recommended for all patients with suspected monogenic phagocytic defects, including chronic granulomatous disease and leukocyte adhesion deficiency.

### Susceptibility to specific infections

Some PIDs present with predisposition to infections with specific organisms or groups of organisms, including severe viral, fungal, or atypical infections. Monogenic immune defects have been identified in an increasing number of these patients, including patients presenting with mucocutaneous candidiasis (34), susceptibility to atypical mycobacteria (35), EBV (36), herpes encephalitis (37) (5–10%), and disseminated vaccine strain measles (such as in IFNAR1 deficiency patients) (38). These conditions can be difficult to diagnose with standard immunology tests, and genomic testing is often required to make a definitive diagnosis. Making a genetic diagnosis of these conditions has important prognostic and treatment implications with targeted therapies available for some conditions.

**Recommendation:** Genomic testing is recommended for patients with susceptibility to specific organisms suggestive of a monogenic IEI.

### Immune dysregulation

Primary immune regulatory disorders (PIRD) are characterized by a loss of normal inflammatory and self-tolerance mechanisms, leading to autoinflammation, autoimmunity (including cytopenias and enteropathy), and lymphoproliferation (39). This group of patients presents a particular diagnostic challenge with variable age of onset, presenting symptoms and disease severity (40). Standard immunological tests are often insufficient to provide a definitive diagnosis for these patients.

There are a number of targeted therapies for patients with PIRD and some patients benefit from early HSCT prior to accumulation of significant end-organ damage (41). There is limited evidence to guide the selection of patients for genomic testing for patients with immune dysregulation.

For patients presenting with autoimmune disease features, including young age of onset, clinical phenotype, inheritance, and the results of functional testing inform the likelihood of a potential monogenic disorder (29).

**Recommendation:** Genomic testing should be considered in patients with immune dysregulation based on clinical features including early age of onset, severity of disease, treatment resistance, and family history.

### Hemophagocytic lymphohistiocytosis

A number of genes are known to be associated with familial or primary hemophagocytic lymphohistiocytosis (HLH) (42). HLH is also a rare complication of some PID. HLH has a high mortality rate, and prompt recognition of patients with genetic predisposition to HLH is essential to provide curative treatment. The likelihood of a genetic diagnosis is highest in infants presenting with HLH (∼60% <1yo, ∼7% aged 12–18) (43).

The likelihood of a genetic diagnosis in adults with HLH is lower, and decisions to proceed with genomic testing should be made in consultation with an adult immunologist or hematologist with expertise in HLH.

**Recommendation:** Rapid genomic testing is recommended for all pediatric patients with suspected familial or primary HLH due to treatment implications.

### Autoimmune lymphoproliferative syndromes

ALPS and ALPS-like disorders present with lymphoproliferation and autoimmunity (most commonly cytopenias). The most common genetic cause of ALPS is the pathogenic variant in the *FAS* gene; however, the spectrum of genetic diseases associated with this presentation is expanding (44, 45).

Genetic diagnoses are increasingly recognized in patients with nonmalignant lymphoproliferation without autoimmunity (∼50%) (46). Genetic diagnosis of these patients has important surveillance and treatment implications, as these conditions are commonly associated with malignancy, and targeted treatments are available.

In the case of a negative result, discussion with a geneticist specialist in regard to somatic testing is recommended.

**Recommendation:** Genomic testing is recommended in all pediatric patients with autoimmune lymphoproliferative syndromes and should be considered in patients with unexplained chronic or recurrent lymphadenopathy, splenomegaly, or organ infiltration by abnormal lymphoid cells.

### Very early onset IBD

Some monogenic immune disorders can present with IBD (47). These patients can present across the lifespan; however, most patients presenting after childhood do not have a genetic basis for their IBD identified (48).The early identification of IBD patients with monogenic disorders is important to guide therapy, with some diagnoses benefiting from early HSCT. Consensus guidelines have been developed to guide the selection of patients with IBD for genomic testing (49).

**Recommendation:** Genomic testing should be considered for all children presenting with IBD <2yo and for patients >2yo with atypical features, including recurrent infections, complex autoimmunity, severe perianal disease, treatment resistance, and family history.

### Evans syndrome

Evans syndrome (autoimmune hemolytic anemia and thrombocytopenia) is associated with monogenic variants in several immune genes in >50% of patients (50, 51). Identification of these monogenic causes is important as targeted therapy is available for some diagnoses.

**Recommendation:** Genomic testing is recommended for all pediatric patients presenting with Evans syndrome.

### Autoinflammation/periodic fever

A number of monogenic causes of autoinflammatory diseases have been described (52). The diagnostic yield from genomic testing these patients is lower (7–20%) than for other IEIs due to a number of factors, including somatic mutations (53, 54). Identification of monogenic diagnosis in these patients can be important to guide therapy; however, treatment options for patients with a range of genetic diagnoses are often the same. Isolated uncomplicated periodic fever in young children (e.g., PFAPA) is considered a complex genetic disorder and rarely monogenic (55).

**Recommendation:** Genomic testing should be considered in selected patients who present with a classical phenotype suggestive of a monogenic disorder or based on atypical features, severity of disease, treatment resistance, and family history, to guide selection of therapy, especially if associated with early age of onset.

## Discussion

Genomic testing is now an essential part of routine clinical immunology care. Consequently, there is need for clear, evidence-based guidelines to support clinical decision-making. This ASCIA guideline provides timely and practical recommendations to assist clinicians in identifying patient, eligible for genomic testing for IEI, and support appropriate test selection and interpretation. The ASCIA guideline aims to enhance diagnostic accuracy, improve patient outcomes, and support equitable access to genomic testing across Australasia and internationally. Nevertheless, further research is still needed to refine patient and test selection strategies, particularly for individuals with varied presentations suggestive of an IEI.

## Materials and methods

### Defining topics and summary of evidence

This guideline was commissioned by the ASCIA immunodeficiency committee, which is comprised of 11 clinical immunologists (adult and pediatric), 5 nurses, and a genetic counsellor. Following an open call to the ASICA immunodeficiency committee members, seven individuals were invited to participate based on content expertise. The committee identified 11 IEI categories to be addressed in this statement based on common clinical presentations, informed by the International Society of Immunodeficiency Society phenotypic classification of IEI (56).

### Literature search and recommendations

An initial PubMed review was conducted by author Peter McNaughton in 2025 for articles reporting on diagnostic yield and clinical features associated with a monogenic diagnosis for each of the 11 IEI categories. Given the heterogeneity of clinical presentations of patients with IEI, the selection of patients for genetic testing remains challenging. Some presenting phenotypes are associated with high rates of monogenic diagnosis, and where this evidence exists, a strong recommendation for genetic testing of all patients was made. For phenotypes with a lower rate of detection of monogenic IEI or limited evidence about yield from genetic testing, more general recommendations to consider clinical features such as age of onset, family history, disease severity, and treatment refractoriness were included.

### Voting statement

Recommendations were made based on interactive email and virtual meeting discussions. The writing group voted on all recommendations, while adding specific comments to the document. A second round of voting was conducted via email with all authors, and 100% consensus was achieved on the final recommendations.

## Data Availability

No new data were generated or analyzed in support of this study.
